# Association of multiple dietary metal intake with cardiovascular-kidney-metabolic syndrome: a cross-sectional study based on NHANES 2003–2018

**DOI:** 10.3389/fnut.2025.1612458

**Published:** 2025-07-21

**Authors:** Sihan Hu, Baojian Wei, Aihua Zhang

**Affiliations:** ^1^School of Public Health, Guangxi Medical University, Nanning, China; ^2^School of Nursing, Shandong First Medical University and Shandong Academy of Medical Sciences, Taian, China

**Keywords:** multiple metal intake, CKM syndrome, NHANES, American, cross-section study

## Abstract

**Background:**

Cardiovascular-kidney-metabolic (CKM) syndrome is a complex condition that encompasses cardiovascular, renal, and metabolic disorders. Dietary metal intake plays a crucial role in maintaining normal physiological functions. This study aims to examine the relationship between dietary intake of multiple metals and CKM syndrome.

**Methods:**

We analyzed data from 15,233 participants aged 20–79 years in the National Health and Nutrition Examination Survey (NHANES) 2003–2018. Dietary metal intake included nine metals: potassium (K), calcium (Ca), magnesium (Mg), phosphorus (P), iron (Fe), copper (Cu), zinc (Zn), and selenium (Se). CKM syndrome was classified into non-advanced (stages 0–2) and advanced (stages 3–4) stages. We employed weighted logistic regression, restricted cubic splines (RCS) regression, weighted quantile sum (WQS) regression, and quantile-based g computation (qgcomp) models to evaluate the associations between individual metal intake and metal intake mixtures with CKM stages. Subgroup analysis was used to explore potential interaction effect between metal intake and other variables.

**Results:**

Weighted logistic regression models showed that Q2 (≤0.80–1.12 mg/d) (OR = 0.74, 95% CI = 0.60, 0.92), Q3 (≤1.12–1.53 mg/d) (OR = 0.74, 95% CI = 0.58, 0.93) and Q4 (>1.53 mg/d) (OR = 0.73, 95% CI = 0.55, 0.95) groups of Cu intake were significantly associated with a reduced incidence of advanced CKM stages compared with Q1 (≤0.80 mg/d) group. The RCS regression models indicated that higher Cu intake was significantly associated with a lower risk of advanced CKM stages (*p* for overall < 0.05). WQS regression and qgcomp models did not reveal significant effect of the mixture. Subgroup analysis found that the effect of Cu was robust in various subgroups.

**Conclusion:**

In conclusion, higher dietary intake Cu was linked to a reduced prevalence of advanced CKM stages in the U. S. adult population.

## 1 Introduction

The Cardiovascular-Kidney-Metabolic (CKM) syndrome, a recently recognized multi-systemic affliction as delineated by the American Heart Association (AHA), involves the complex interactions between obesity, diabetes mellitus, chronic renal conditions, and cardiovascular illnesses (1, 2). This syndrome significantly influences patient outcomes and is associated with elevated mortality rates that surpass the aggregate risks of each individual condition (3). In the United States, the incidence of CKM is escalating. Information gleaned from the National Health and Nutrition Examination Survey (NHANES) reveals that nearly 90% of the adult population in the US fulfill the criteria for CKM stage 1 or beyond, with roughly 15% being categorized under the advanced stages of CKM (stages 3 or 4) (4). Therefore, it is particularly important to find effective measures to prevent CKM syndrome.

Metal elements, including constant and trace metals, are key nutrients for maintaining normal physiological functions of the body (5, 6). Previous studies have shown that metals are closely related to cardiovascular, renal, and metabolic processes. Calcium exerts its influence on the susceptibility to cardiovascular disease (CVD) via a variety of pathways, encompassing modulation of serum cholesterol levels, insulin release, and insulin sensitivity, as well as impacting vasodilation, adiposity, and vascular calcification. A deficiency in zinc and copper may elevate the risk of developing coronary artery disease (7). In addition, dietary zinc and selenium intake can significantly reduce the risk of chronic kidney disease (CKD) in adults (8, 9). Magnesium, phosphorus and selenium may have beneficial effects on lipid metabolism (10, 11). CKM syndrome involves complex interactions between metabolism, CKD, and cardiovascular disease, and it is unclear what role metals play in CKM syndrome. Importantly, previous studies have mostly focused on single metals, and the interactions between metals may affect each other’s physiological functions (12, 13).

To address this gap, we conducted a large-scale cross-sectional study using the National Health and Nutrition Examination Survey (NHANES) data to investigate the association between multiple metal intakes in American adults and CKM syndrome. We hope to reveal the association between dietary metal mixtures and CKM and provide clues from a dietary perspective for the prevention and treatment of CKM syndrome.

## 2 Methods

### 2.1 The study design and population

The NHANES is an extensive research initiative conducted by the Centers for Disease Control and Prevention (CDC) in the United States to evaluate the health condition and associated health determinants among the U. S. population. Furthermore, the programs of NHANES have been sanctioned by the ethical review board of the National Center for Health Statistics (NCHS) (14).

**Figure 1:**
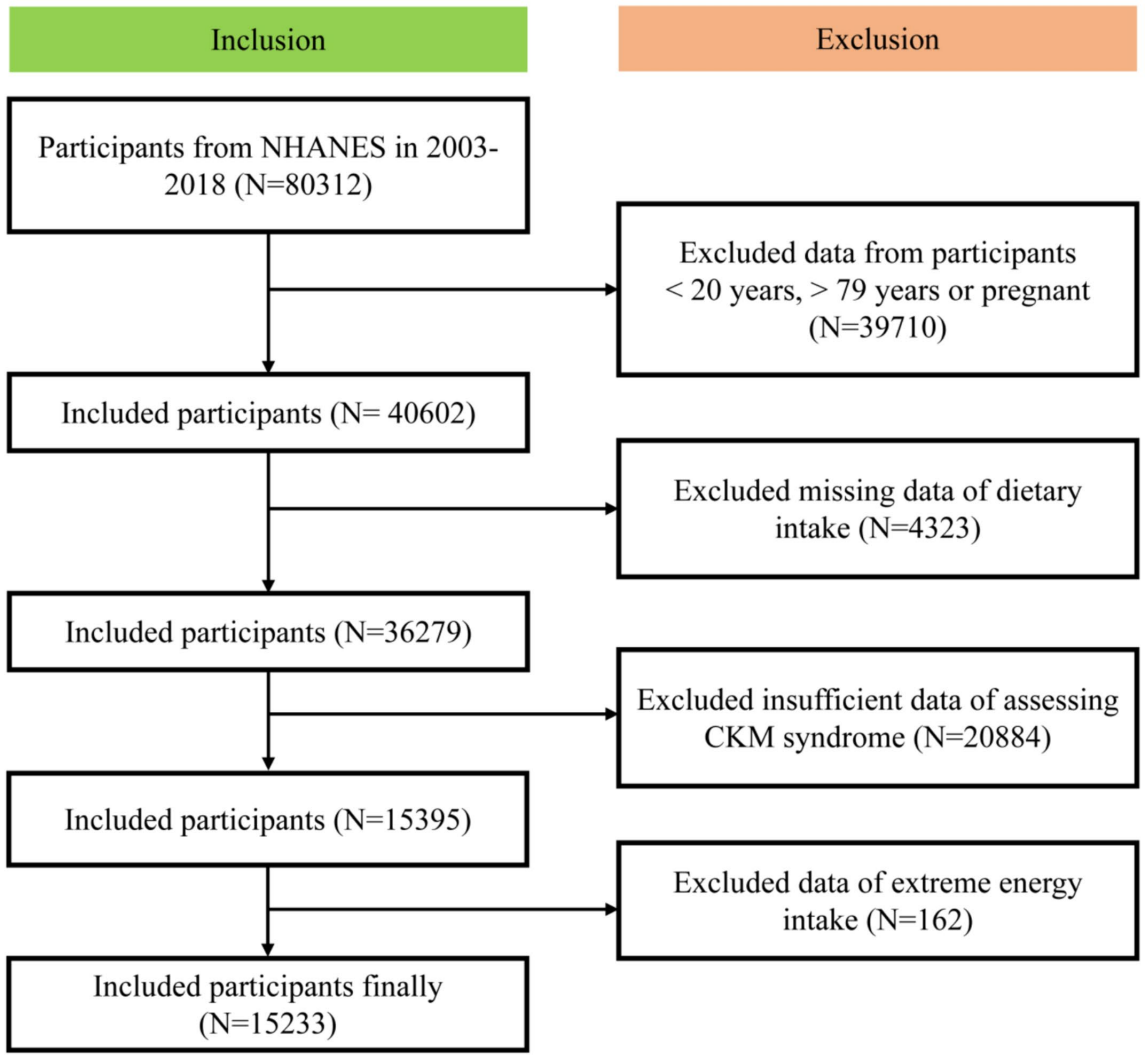
Screening flow of participants.

In this study, we included participants from the NHANES between 2003 and 2018. The exclusion criteria were: under 20 years old, over 79 years old, pregnant, missing dietary intake data, with insufficient data for assessing CKM syndrome and with extreme energy intake data. Extreme energy intake was total energy intake < 500 kcal/day or > 5,000 kcal/day for females or <500 kcal/day or > 8,000 kcal/day males (15). Ultimately, our analysis included a final cohort of 15,233 participants (Figure 1).

### 2.2 Assessment of CKM syndrome

The AHA has released diagnostic criteria for CKM syndrome, but the indicators involved in this criterion cannot be fully found in the NHANES database. Therefore, we referred to the diagnostic criteria applicable to the NHANES database mentioned in previous literature, which are very similar to those proposed by the AHA (4). Overall, CKM syndrome consists of 5 stages (stages 0 to 4), divided according to each individual’s obesity, cardiovascular, renal, and metabolic status. Please refer to Supplementary Table 1 for detailed diagnostic criteria.

Complex calculated indicators included the estimated glomerular filtration rate (eGFR) and 10-year cardiovascular disease risk. The eGFR was calculated using the 2021 race-and ethnicity-free Chronic Kidney Disease Epidemiology Collaboration creatinine equation which included serum creatinine and sex (16). And the 10-year CVD risk was assessed using prediction equations for absolute risk of total CVD, which included factors such as sex, age, blood lipids, blood pressure, diabetes, smoking status, eGFR, and the use of lipid-lowering and antihypertensive medications (17). High CVD risk was operationally defined as a ≥ 20% 10-year CVD risk. Please refer to Supplementary Table 2 for detailed calculation process. CKD stage was determined by eGFR and (Urine Albumin-to-Creatinine Ratio) UACR (18). We defined the CKM stage as a binary variable, with 0, 1, or 2 being nonadvanced CKM stages and stages 3 or 4 being advanced CKM stages (19).

### 2.3 Dietary metal intake

Dietary intakes were from a 24-h dietary review survey. Trained investigators conduct interviews with respondents, with the first dietary survey conducted in person and the second conducted through telephone interviews 3 to 10 days after the first survey. Considering the accuracy and authenticity of face-to-face interview data, we only included data from the first dietary survey (20). In this study, we included 9 types of dietary metal intake, including potassium (K), calcium (Ca), magnesium (Mg), phosphorus (P), iron (Fe), copper (Cu), zinc (Zn), and selenium (Se).

### 2.4 Covariates

Covariates encompassed a spectrum of demographic, lifestyle factors and energy intake. Demographic factors comprised gender (male, female), age (20–39, 40–59, and 60 years and above), ethnicity (Mexican American, other Hispanic, non-Hispanic White, non-Hispanic Black, and other race including multi-racial), educational attainment (below high school, high school graduate/GED or equivalent, and above high school), household poverty-to-income ratio (PIR) (≤1.3, 1.3–3.5, and above 3.5), and marital status (married/living with partner, widowed/divorced/separated, and unmarried). Lifestyle factors included smoking status, drinking status, and physical exercise. Participants were also classified into nonsmokers, former smokers, and current smokers based on their smoking habits. The status of alcohol intake was ascertained by the quantity of alcohol consumed last year, including nondrinkers (no alcohol consume), moderate drinkers (≤ 2 drinks/day for male, ≤ 1 drinks/day for female), heavy drinkers (> 2 drinks/day for male, > 1 drinks/day for female) (21). Low physical activity was defined as no moderate or vigorous physical activity in a typical week from self-reporting (1). The daily energy intake came from the dietary survey on the first day.

### 2.5 Statistical analysis

All statistical models were completed using R (version 4.4.1). Two-tailed *p* values < 0.05 were considered statistically significant. Given the multi-stage sampling methodology employed in NHANES, we utilized the dietary survey weight “WTDRD1” to stand for the U. S. population (22). In the descriptive statistical analysis, continuous variables, encompassing energy intake and 9 dietary metal intakes, were depicted through weighted medians (25th, 75th), while categorical variables were exhibited as counts (weighted percentages). Participants were grouped into two categories: non-advanced stages and advanced stages. We contrasted the distribution differences of the variables between these two groups, employing weighted Kruskal-Wallis tests for continuous variables and weighted chi-square tests for categorical variables (23). Spearman rank correlation analysis was implemented to appraise the correlation of dietary metal intake (24). To address the missing values of covariates, aiming to optimize the sample size to the fullest extent, we applied multiple imputation techniques in the “MICE” package (25).

To investigate the relationship between individual dietary metal intake and advanced CKM stages, we applied weighted logistic regression. Each dietary metal intake was stratified into four groups based on its quartiles (Q1-Q4), with the Q1 group serving as a reference point (26). Three models were constructed in this segment: Model 1, the unadjusted model; Model 2, adjusted for demographic variables; Model 3, adjusted for all covariates. Restricted cubic splines (RCS) regression model according to the quantity of knots that meet the minimal Akaike information criterion (AIC) each dietary metal intake was applied to evaluate the potential nonlinear relationship between dietary metal intake and advanced CKM stages (27).

Weighted Quantile Sum (WQS) regression model is extensively utilized in environmental epidemiology to explore the impacts of pollutant mixtures on health outcomes. We substituted environmental pollutants with dietary metal intake and assessed the impact of a mixture of nine dietary metal intakes on advanced CKM stages. In essence, WQS regression categorized various dietary metal intakes into quantiles and computed a weighted index represents the combined effects of the mixture of 9 dietary metal intakes. Additionally, WQS regression established two models by hypothesizing a positive or negative correlation between the mixture and the outcome (28). Specifically, each dietary metal intake will possess a corresponding weight denoting its contribution to advanced CKM stages in each model. If the weight of a dietary metal intake surpasses 0.11 (1/9), it is deemed to play a predominant role in the mixture. The dataset was randomly divided into a training set (50%) and a validation set (50%), and was sampled 1,000 times to achieve more precise results. We also established a Quantile-based computation (gqcomp) model to substantiate the outcomes of WQS regression. This method integrates the straightforward inference of WQS regression with the adaptability of g-computation (29). Unlike WQS regression, the qgcomp model does not presuppose a positive or negative correlation between the mixture and the outcome separately. It also generates a weight for each component to articulate the significance of each component within the mixture. The WQS regression and qgcomp models are not yet suitable for weighted analysis of NHANES (30).

Ultimately, we established subgroup analyses to assess the potential interaction between the dietary metal mixture and subgroup variables including age, sex, smoking status, drinking status, physical activity and energy intake (Classified by median calorie count).

## 3 Results

### 3.1 Study population and dietary metal intake

The basic characteristics of 15,233 participants were displayed in Table 1. 12,931 (approximately 88.2%) were in nonadvanced stages of CKM and 2,302 participants (approximately 11.8%) were in advanced stages of CKM. Moreover, about half of the participants (50.2%) were female. By comparing the differences in the distribution of different demography and lifestyle variables between the two groups, we found that there were significant differences between the two groups in all the variables. In terms of metal intake, participants in the advanced stage might have lower levels of all metal intakes (All *p* < 0.05).

**Table 1:** Characteristics and dietary metal intakes of participants grouped by CKM stages in NHANES 2003–2018.

Variables	Overall	Nonadvanced stages group	Advanced stages group	*P*
n/n (%)	15,233	12,931 (88.2)	2,302 (11.8)	
Age, *n* (%)				<0.001
20–39 years	5,256 (36.4)	5,150 (40.6)	106 (5.3)	
40–59 years	5,450 (39.8)	4,976 (41.8)	474 (24.9)	
≥60 years	4,527 (23.8)	2,805 (17.6)	1722 (69.9)	
Gender, *n* (%)				<0.001
Male	7,679 (49.8)	6,323 (48.7)	1,356 (57.5)	
Female	7,554 (50.2)	6,608 (51.3)	946 (42.5)	
Race, *n* (%)				<0.001
Mexican American	2,600 (8.4)	2,304 (8.8)	296 (5.3)	
Other Hispanic	1,467 (5.4)	1,269 (5.6)	198 (4)	
Non-Hispanic White	6,511 (68.2)	5,413 (67.9)	1,098 (70.9)	
Non-Hispanic Black	3,112 (10.6)	2,553 (10.3)	559 (13.1)	
Other Race–Including Multi-Racial	1,543 (7.4)	1,392 (7.5)	151 (6.7)	
Education, *n* (%)				<0.001
Less than high school	3,679 (15.4)	2,923 (14.3)	756 (23.6)	
High school graduate/GED or equivalent	3,478 (23.7)	2,908 (23.3)	570 (26.6)	
Higher than high school	8,076 (60.9)	7,100 (62.4)	976 (49.8)	
PIR, *n* (%)				<0.001
≤1.3	4,634 (21.8)	3,831 (21.2)	803 (26.4)	
1.3–3.5	5,784 (35.4)	4,817 (34.6)	967 (41.1)	
>3.5	4,815 (42.8)	4,283 (44.2)	532 (32.5)	
Marital status, *n* (%)				<0.001
Married/living with partner	9,409 (64)	8,017 (64)	1,392 (64.3)	
Widowed/divorced/separated	3,016 (17.4)	2,279 (15.8)	737 (29.3)	
Never married	2,808 (18.6)	2,635 (20.2)	173 (6.4)	
Smoking status, *n* (%)				<0.001
Non smokers	8,239 (52.7)	7,358 (54.9)	881 (36.6)	
Former smokers	3,669 (25.2)	2,791 (23.5)	878 (37.9)	
Current smokers	3,325 (22)	2,782 (21.6)	543 (25.4)	
Drinking status, *n* (%)				<0.001
Non drinkers	5,053 (27.6)	3,972 (25.6)	1,081 (42.5)	
Moderate drinkers	9,150 (64.2)	8,044 (65.9)	1,106 (51.7)	
Heavy drinkers	1,030 (8.2)	915 (8.5)	115 (5.7)	
Physical activity, *n* (%)				<0.001
High	11,225 (77.4)	9,834 (79.3)	1,391 (63.7)	
Low	4,008 (22.6)	3,097 (20.7)	911 (36.3)	
Energy intake [kal, median (25th, 75th)]	2044.00 (1529.01, 2697.00)	2082.00 (1559.00, 2741.00)	1800.60 (1365.00, 2337.00)	<0.001
Dietary metal intakes, median (25th, 75th)				
K, mg/d	2565.66 (1874.00, 3381.00)	2585.42 (1884.12, 3403.00)	2450.87 (1779.77, 3217.01)	<0.001
Ca, mg/d	845.19 (560.63, 1241.00)	858.00 (569.00, 1254.18)	757.06 (493.88, 1096.53)	<0.001
Mg, mg/d	280.00 (204.00, 377.00)	284.00 (206.00, 382.00)	255.00 (183.00, 344.00)	<0.001
Na, mg/d	3321.00 (2381.00, 4445.26)	3355.00 (2414.00, 4495.00)	3065.98 (2139.95, 3981.34)	<0.001
P, mg/d	1297.00 (947.00, 1747.00)	1320.00 (962.00, 1771.24)	1162.71 (846.32, 1533.48)	<0.001
Fe, mg/d	13.38 (9.47, 18.94)	13.51 (9.55, 19.09)	12.47 (8.97, 17.84)	<0.001
Cu, mg/d	1.16 (0.83, 1.59)	1.18 (0.85, 1.61)	1.05 (0.73, 1.45)	<0.001
Zn, mg/d	10.33 (7.15, 14.81)	10.46 (7.23, 14.95)	9.31 (6.50, 13.85)	<0.001
Se, mcg/d	105.82 (73.20, 144.50)	107.20 (74.60, 146.90)	95.08 (65.50, 128.40)	<0.001

Supplementary Figure 1 showed the correlation heatmap of 9 metal intakes. All the metal intakes are positively correlated. The highest correlation was observed between Mg and Cu (Correlation coefficient = 0.85).

### 3.2 Associations between single metal intake and advanced CKM stages

Table 2 showed the associations between single metal intake and advanced CKM stages in weighted logistic regression models. The population was grouped into Q1-Q4 according to each metal intake. In the unadjusted model, K (Q4), Ca (Q3, Q4), Mg (Q2, Q3, Q4), Na (Q2, Q3, Q4), P (Q3, Q4), Fe (Q3, Q4), Cu (Q2, Q3, Q4), Zn (Q3, Q4) and Se (Q2, Q3, Q4) were negative with advanced CKM stages compared with Q1. After adjusting for demography variables, we found that K (Q4), Ca (Q4), Mg (Q2, Q4), Na (Q2, Q3, Q4), P (Q3, Q4), Fe (Q4), Cu (Q2, Q3, Q4), Zn (Q3, Q4) and Se (Q2, Q3, Q4) were still negative associations with advanced CKM stages compared with Q1. In the fully-adjusted model, only Q2 (OR = 0.74, 95% CI = 0.60, 0.92), Q3 (OR = 0.74, 95% CI = 0.58, 0.93) and Q4 (OR = 0.73, 95% CI = 0.55, 0.95) groups of Cu intake remain negative associations with advanced CKM stages compared with Q1 group (All *p* < 0.05).

**Table 2:** The associations of single metal intake with advanced CKM stages in weighted logistic regression models.

Dietary metal intakes	Model 1	Model 2	Model 3
	OR (95% CI)	OR (95% CI)	OR (95% CI)
K			
Q1 (≤1766 mg/d)	Reference	Reference	Reference
Q2 (1766-2439mg/d)	0.93 (0.79, 1.10)	0.82 (0.67, 1.01)	0.98 (0.80, 1.20)
Q3 (2439-3286mg/d)	0.90 (0.77, 1.04)	0.83 (0.69, 1.00)	1.08 (0.89, 1.31)
Q4 (>3,286 mg/d)	0.72 (0.59, 0.89)	0.69 (0.55, 0.88)	1.04 (0.78, 1.38)
Ca			
Q1 (≤512 mg/d)	Reference	Reference	Reference
Q2 (512-792mg/d)	0.83 (0.69, 1.00)	0.87 (0.70, 1.09)	1.02 (0.81, 1.29)
Q3 (792-1157mg/d)	0.72 (0.59, 0.87)	0.8 (0.64, 1.01)	1.02 (0.79, 1.32)
Q4 (>1,157 mg/d)	0.57 (0.47, 0.70)	0.77 (0.62, 0.97)	1.12 (0.86, 1.47)
Mg			
Q1 (≤195 mg/d)	Reference	Reference	Reference
Q2 (195-268mg/d)	0.76 (0.65, 0.89)	0.8 (0.66, 0.98)	0.92 (0.74, 1.14)
Q3 (268-365mg/d)	0.74 (0.60, 0.91)	0.91 (0.71, 1.17)	0.92 (0.73, 1.15)
Q4 (>365 mg/d)	0.51 (0.42, 0.61)	0.73 (0.58, 0.91)	0.89 (0.64, 1.24)
Na			
Q1 (≤2,258 mg/d)	Reference	Reference	Reference
Q2 (2258-3197mg/d)	0.77 (0.66, 0.92)	0.78 (0.64, 0.96)	0.93 (0.76, 1.15)
Q3 (3197-4369mg/d)	0.69 (0.59, 0.82)	0.72 (0.59, 0.87)	1.24 (0.95, 1.60)
Q4 (>4,369 mg/d)	0.55 (0.45, 0.68)	0.61 (0.48, 0.77)	1.16 (0.89, 1.53)
P			
Q1 (≤897 mg/d)	Reference	Reference	Reference
Q2 (897-1237mg/d)	0.87 (0.74, 1.03)	0.92 (0.76, 1.11)	1.08 (0.88, 1.33)
Q3 (1237-1679mg/d)	0.62 (0.52, 0.73)	0.68 (0.56, 0.83)	0.91 (0.72, 1.16)
Q4 (>1,679 mg/d)	0.48 (0.40, 0.58)	0.64 (0.51, 0.80)	0.98 (0.69, 1.38)
Fe			
Q1 (≤9.13 mg/d)	Reference	Reference	Reference
Q2 (9.13–13.04 mg/d)	0.92 (0.78, 1.09)	0.96 (0.79, 1.17)	1.11 (0.90, 1.36)
Q3 (13.04–18.52 mg/d)	0.78 (0.64, 0.94)	0.83 (0.67, 1.03)	1.10 (0.86, 1.39)
Q4 (>18.52 mg/d)	0.72 (0.59, 0.86)	0.78 (0.63, 0.98)	1.13 (0.87, 1.47)
Cu			
Q1 (≤0.80 mg/d)	Reference	Reference	Reference
Q2 (0.80–1.12 mg/d)	0.69 (0.59, 0.80)	0.66 (0.54, 0.80)	0.74 (0.60, 0.92)
Q3 (1.12–1.53 mg/d)	0.65 (0.54, 0.78)	0.61 (0.49, 0.76)	0.74 (0.58, 0.93)
Q4 (>1.53 mg/d)	0.49 (0.42, 0.59)	0.54 (0.45, 0.65)	0.73 (0.55, 0.95)
Zn			
Q1 (≤6.80 mg/d)	Reference	Reference	Reference
Q2 (6.80–9.84 mg/d)	0.88 (0.73, 1.06)	0.92 (0.71, 1.18)	1.10 (0.86, 1.42)
Q3 (9.84–14.22 mg/d)	0.68 (0.58, 0.81)	0.74 (0.62, 0.88)	0.96 (0.78, 1.17)
Q4 (>14.22 mg/d)	0.67 (0.56, 0.82)	0.80 (0.64, 0.99)	1.18 (0.90, 1.56)
Se			
Q1 (≤71.10 mg/d)	Reference	Reference	Reference
Q2 (71.10–103.20 mg/d)	0.72 (0.61, 0.86)	0.73 (0.58, 0.90)	0.82 (0.65, 1.03)
Q3 (103.20–142.70 mg/d)	0.68 (0.58, 0.80)	0.81 (0.66, 0.99)	1.02 (0.81, 1.28)
Q4 (>142.70 mg/d)	0.48 (0.39, 0.58)	0.61 (0.49, 0.77)	0.87 (0.65, 1.17)

Model 1 was not adjusted for any covariate. Model 2 was adjusted for age, gender, race, education, PIR and marital status. Model 3 was adjusted for all covariates.

**Figure 2:**
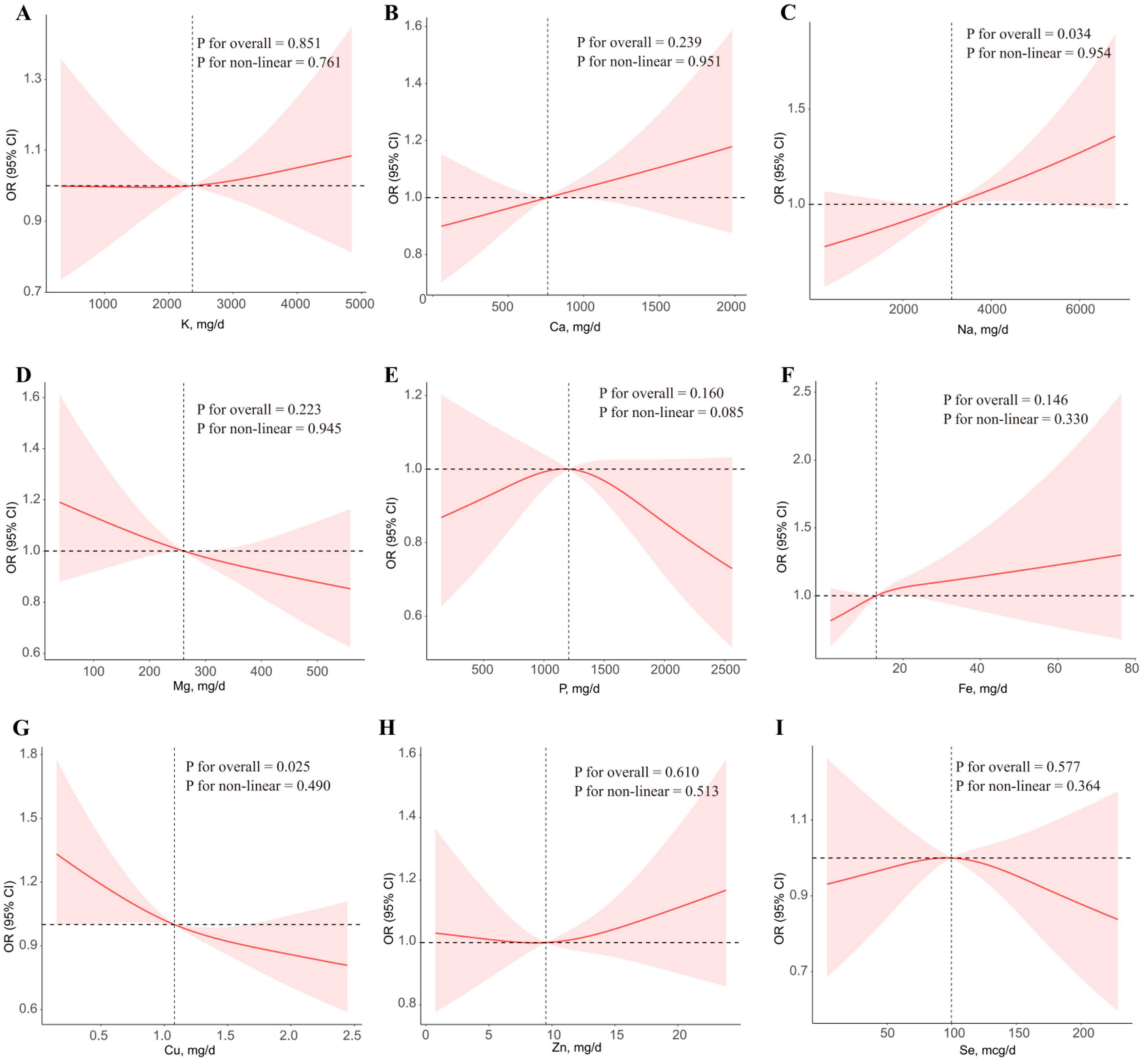
The associations of single metal intake with advanced CKM stages in RCS regression models. **(A)** K, **(B)** Ca, **(C)** Mg, **(D)** Na, **(E)** P, **(F)** Fe, **(G)** Cu, **(H)** Zn, **(I)** Se. All the models were adjusted for all covariates.

The results of RCS regression models were displayed in Figure 2. After adjusting for all covariates, higher intake of Cu was linearly associated with lower risk of advanced CKM stages (*p* for overall < 0.05 and *P* for non-linear > 0.05). However, higher intake of Na was linearly associated with higher risk of advanced CKM stages (*p* for overall < 0.05 and *P* for non-linear > 0.05). No significant association was found between the intake of other metals and advanced CKM stages (All *p* for overall > 0.05).

### 3.3 Associations between vitamin intake mixture and advanced CKM stages

**Figure 3:**
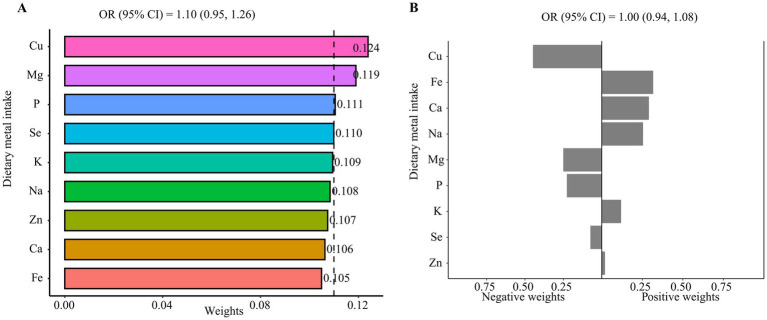
The associations of metal intake mixture with advanced CKM stages. **(A)** WQS regression model, **(B)** qgcomp model. All the models were adjusted for all covariates.

Subsequently, the associations between the metal intake mixture and advanced CKM stages were examined using WQS regression and qgcomp models (Figure 3). Due to the single metal intake model indicating a significant negative correlation between copper intake and advanced CKM stages, we firstly hypothesized a negative relationship between metal intake mixture and advanced CKM stages. However, we did not find a significant effect of metal ingestion mixtures (OR: 1.10, 95% CI: 0.95, 1.26). Conversely, when we hypothesized a positive relationship between metal intake mixture and advanced CKM stages, no significant effect was observed either (OR: 1.13, 95% CI: 0.98, 1.29; Supplementary Figure 2). The qgcomp model yielded similar results to the WQS regression, corroborating an insignificant effect of metal ingestion mixtures (OR: 1.00, 95% CI: 0.94, 1.08; Figure 3B). Although the overall effect of multiple metal intake was not significant, copper intake seemed to be associated with a low risk of advanced CKM stages.

### 3.4 Subgroup analysis of dietary cu intake in relation to advanced CKM stages

We further evaluated the potential interaction between the Cu intake and subgroup variables, including age, sex, smoking status, drinking status, physical activity and energy intake (Table 3). The association between Cu intake and advanced CKM stages was robust across subgroups. In some subgroups, we still found a correlation between groups of high copper intake and lower risk of advanced CKM stages compared with the lowest Cu intake group.

**Table 3:** Subgroup analysis between the Cu intake and subgroup variables.

Characteristic	OR (95% CI)				*p* for interaction
	Q1	Q2	Q3	Q4	
Age					0.330
20-39 years	Reference	0.54 (0.25,1.15)	0.86 (0.38,1.96)	0.73 (0.23,2.36)	
40-59 years	Reference	0.66 (0.46,0.95)	0.73 (0.48,1.13)	0.55 (0.33,0.91)	
≥60 years	Reference	0.84 (0.64,1.11)	0.76 (0.56,1.03)	0.91 (0.64,1.28)	
Gender					0.066
Male	Reference	0.77 (0.58, 1.03)	0.93 (0.65, 1.32)	0.79 (0.53, 1.17)	
Female	Reference	0.72 (0.55, 0.95)	0.55 (0.40, 0.76)	0.70 (0.48, 1.02)	
Smoking status					0.561
Non smokers	Reference	0.63 (0.45, 0.87)	0.54 (0.41, 0.73)	0.57 (0.37, 0.87)	
Former smokers	Reference	0.88 (0.60, 1.30)	0.87 (0.56, 1.35)	0.85 (0.56, 1.29)	
Current smokers	Reference	0.78 (0.53, 1.17)	0.97 (0.58, 1.61)	0.91 (0.52, 1.60)	
Drinking status					0.227
Non drinkers	Reference	0.89 (0.67, 1.18)	0.66 (0.43, 1.00)	0.65 (0.41, 1.02)	
Moderate drinkers	Reference	0.72 (0.53, 0.96)	0.83 (0.61, 1.12)	0.84 (0.59, 1.19)	
Heavy drinkers	Reference	0.37 (0.15, 0.94)	0.49 (0.14, 1.65)	0.33 (0.11, 1.03)	
Physical activity					0.540
High	Reference	0.69 (0.52, 0.91)	0.75 (0.55, 1.00)	0.70 (0.51, 0.96)	
Low	Reference	0.82 (0.59, 1.14)	0.68 (0.47, 0.99)	0.76 (0.46, 1.26)	
Energy intake					0.339
≤ 1982kal	Reference	0.79 (0.61, 1.02)	0.69 (0.49, 0.96)	0.79 (0.55, 1.11)	
> 1982kal	Reference	0.50 (0.28, 0.89)	0.62 (0.35, 1.09)	0.57 (0.31, 1.05)	

All the models were adjusted for all covariates.

## 4 Discussion

In a word, we utilized the data of the NHANES 2003–2018 and established a large-scale cross-sectional study and examine the association between dietary metal intake and CKM syndrome among American adults. We used weighted logistic regression model, RCS regression model, WQS regression model, and qgcomp model to assess individual and combined effects of metal intake comprehensively. Weighted logistic regression models showed that Q2 (≤0.80–1.12 mg/d), Q3 (≤1.12–1.53 mg/d) and Q4 (>1.53 mg/d) groups of Cu intake were significantly associated with a reduced incidence of advanced CKM stages compared with Q1 (≤0.80 mg/d) group. The RCS regression models indicated that higher Cu intake was significantly associated with a lower risk of advanced CKM stages. The two mixture models did not reveal a significant effect of metal ingestion mixtures. Subgroup analysis revealed the robust association between copper intake and advanced CKM stages across subgroups. In summary, the intake of multiple metal mixture by American adults may not be associated with the risk of advanced CKM stages.

CKM syndrome is an emerging disease that encompasses complex associations between the cardiovascular, renal, and metabolic systems. In this study, we identified the correlation of higher Cu intake and lower risk of CKM syndrome risk. A study from the NHANES showed that dietary copper intake was significantly negatively correlated with the incidence rate of CVD (31). Copper is closely related to the metabolic system. A randomized controlled trial showed that participants’ fasting blood glucose levels and insulin resistance improved with an increase in copper intake (32). In addition, high copper exposure in early pregnancy can significantly increase the level of high-density lipoprotein cholesterol (HDL-C) in Chinese pregnant women (33). Conversely, Urinary copper may lead to the development of abnormal blood lipids (34). Abnormal copper metabolism plays a key role in the progression of CKD (35). In a cross-sectional survey of 2,210 adults in China, high concentration of serum Cu was significantly associated with high-risk CKD (36). However, a large prospective cohort study showed a U-shaped association between dietary copper intake and CKD incidence 30 years later (37). In summary, a large amount of literature has confirmed the correlation between copper and multiple systems in the human body, although research conclusions are inconsistent. Firstly, there may be differences in dietary copper intake and copper concentration among different biological samples. Secondly, differences in conclusions are caused by research populations from different regions or ethnicities. Thirdly, research design, statistical methods, and sample size may all lead to statistical differences in the results. Most importantly, CKM syndrome is a syndrome that combines three systems, not just one of the cardiovascular, metabolic, or renal systems and. We have discovered for the first time the association between dietary copper intake and this complex disease, and this conclusion requires further research to confirm.

Although we did not find significant associations between other metals and CKM syndrome, previous literature has reported their relationships with cardiovascular, renal, and metabolic systems. Maintaining normal levels of P intake may be beneficial for CVD and CKD (38). A meta-analysis showed elevated levels of dietary Mg or serum Mg were linearly and negatively correlated with the risk of total CVD events (39). Kuria et al. found that compared with the low Se state in the body, the incidence of CVD was lower under physiological high Se state (40). High Se exposure may also lower total cholesterol (TC) and increase HDL-C (41). K was the most abundant cations in intracellular fluids and play a decisive role in maintaining normal cellular function (42). Higher K levels may lower the risk of CVD by lowering blood pressure. The main way for the human body to ingest Na is through table salt (43). Due to insufficient human control over salt intake, the current research results indicate that strict Na intake is crucial for human health. However, the importance of low Na levels in maintaining human health cannot be ignored (44). In addition, Ca and Zn have also shown beneficial effects on the cardiovascular and renal systems (45, 46). Similarly, the inconsistency between the above conclusions and our research findings may be attributed to the complexity of CKM syndrome compared to a single systemic disease, study design, study population, and statistical methods.

After Cu enters the human body, it is mainly absorbed by the duodenum and small intestine, and then secreted into the bloodstream to combine with various soluble substances. Then Cu was transported to different organs to exert its effects (47). The mechanism by which Cu affect the process of CKM syndrome is still unclear. We speculate that inflammation and oxidative stress are common influencing factors of the cardiovascular, renal, and metabolic systems. The antioxidant and anti-inflammatory effects of Cu seem to have become a consensus. Experimental studies have shown that dietary Reduce NF-κB and IRF3 activation in macrophages stimulated by LPS (48). Moreover, Cu is a cofactor of superoxide dismutase (SOD) and can regulate the activity of SOD (49). On the other hand, the close relationship between aging and chronic diseases has been reported in literature. An epidemiological study reveals that dietary Cu intake obviously reduced aging (50). Further research is needed to explore the potential mechanisms underlying the beneficial effects of copper on CKM syndrome.

We applied advanced mixed exposure models to investigate the comprehensive effects of multiple metal ingestion on CKM syndrome. It is basically impossible to consume only one metal under a normal dietary pattern, as the nutrients in food are diverse (51). Our design conforms to the real dietary patterns of humans and fills the gap in research on single metal intake. In addition, there are interactions between different metal elements or other nutrients, and relying solely on a single variable model may not accurately identify their effects on the human body. Therefore, we call on researchers to pay more attention to the intake of multiple nutrients in future studies. In the mixture model used in this study, we did not determine whether dietary metal mixtures have a significant positive or negative effect on CKM syndrome. This may be due to the cancelation of positive or negative effects caused by the intake of different metals, as demonstrated by the RCS model.

Our research has several advantages. Firstly, this is the first study to explore the association between dietary metal intake mixtures and an emerging disease called CKM syndrome. Our research provided dietary recommendations for the prevention and treatment of CKM syndrome. Secondly, we utilized reliable mixed exposure models to identify the dominant metal ingestion. Thirdly, the large sample size of this study ensured the authenticity of the results. The shortcomings of this study cannot be ignored. Firstly, short-term dietary survey may not fully reflect the long-term dietary habits of participants. However, numerous studies have confirmed the reliability of short-term dietary surveys (52). Secondly, the staging criteria for CKM syndrome in NHANES have not been unified yet, which may have a certain impact on the results. We compared other literature and found that the difference in staging criteria is negligible. Finally, cross-sectional studies have less ability to validate causal associations compared to longitudinal studies. Another key point is that we did not account for the potential influence of other dietary nutrients, foods, or dietary patterns because of potential collinearity. In the future, new statistical methods such as machine learning may be reused to comprehensively explore the role of nutrients in CKM syndrome. We believe that more research will confirm our conclusions in the future.

## 5 Conclusion

Our findings indicated that higher dietary Cu (>1.53 mg/d) was associated with a lower prevalence of advanced CKM stages compared lower dietary Cu in the U. S. adult population. However, multiple dietary metal intakes did not show a significant effect on advanced CKM stages. Further experimental and longitudinal cohort studies are warranted to corroborate these observations.

## Data Availability

The original contributions presented in the study are included in the article/Supplementary material, further inquiries can be directed to the corresponding author/s.
