# Supplementary material for: Association of multiple dietary metal intake with cardiovascular-kidney-metabolic syndrome: a cross-sectional study based on NHANES 2003–2018
Source: Front Nutr. 2025 Jul 21;12:1612458. doi: 10.3389/fnut.2025.1612458 (PMC12320698; doi:10.3389/fnut.2025.1612458)
Supplement: Supplementary file 1 [file Table_1.docx]

Supplement Table 1. Staging criteria for CKM syndrome.

| CKM stages | Staging criteria |
| --- | --- |
| Stage 0 | Participants with a body mass index (BMI; calculated as weight in kilograms divided by height in meters squared) of 18.5 to 24.9 and waist circumference of less than 88 cm for women or less than 102 cm for men who did not meet criteria for the other stages |
| Stage 1 | Participants with a BMI of 25 or higher, a waist circumference of 88 cm or greater for females or 102 cm or greater for males, or prediabetes (fasting blood glucose of 100-124 mg/dL, glycosylated hemoglobin A1c of 5.7%-6.4%, or taking diabetes medications [to convert glucose to mmol/L, multiply by 0.0555; to convert hemoglobin A1c to proportion of total hemoglobin, multiply by 0.01]). |
| Stage 2 | Participants with metabolic risk factors (elevated fasting serum triglyceride levels 135 mg/dL [to convert to mmol/L, multiply by 0.0113], hypertension, diabetes, or metabolic syndrome) or moderate- to high-risk CKD according to Kidney Disease: Improving Global Outcomes criteria |
| Stage 3 | Participants with very-high-risk CKD by Kidney Disease: Improving Global Outcomes criteria or a high predicted 10-year CVD risk |
| Stage 4 | Participants with self-reported CVD (coronary heart disease, angina, heart attack, heart failure, or stroke) |

Supplement Table 2. Calculation process for 10-year CVD risk.

| **Total CVD (Base 10 year)** | |
| --- | --- |
| **Women** | **log-Odds** = -3.307728 + 0.7939329 × (age – 55) /10 + 0.0305239 × ((TC – HDL) × 0.02586 – 3.5) – 0.1606857 × (HDL × 0.02586 – 1.3) /0.3 – 0.2394003 × (min(SBP, 110) – 110) /20 + 0.360078 × (max(SBP, 110) – 130) /20 + 0.8667604 × (if diabetes) + 0.5360739 × (if current smoker) + 0.6045917 × (min(eGFR, 60) – 60) / -15 + 0.0433769 × (max(eGFR, 60) – 90) / -15 + 0.3151672 × (if using anti-hypertensive medication) – 0.1477655 × (if using statin) – 0.0663612 × (if using anti-hypertensive medication) × (max(SBP, 110) – 130) /20 + 0.1197879 × (if using statin) × ((TC – HDL) × 0.02586 – 3.5) – 0.0819715 × (age – 55) /10 × ((TC – HDL) × 0.02586 – 3.5) + 0.0306769 × (age – 55) /10 × (HDL × 0.02586 × 0.02586 – 1.3) /0.3 – 0.0946348 × (age – 55) /10 × (max(SBP, 110) – 130) /20 – 0.27057 × (age – 55) /10 × (if diabetes) – 0.078715 × (age – 55) /10 × (if current smoker) – 0.1637806 × (age – 55) /10 × (min(eGFR, 60) – 60) / -15  **Risk** = exp(log-Odds) / (1 + exp(log-Odds)) |
| **Men** | **log-Odds** = -3.031168 + 0.7688528 × (age – 55) /10 + 0.0736174 × ((TC – HDL) × 0.02586 – 3.5) – 0.0954431 × (HDL × 0.02586 – 1.3) /0.3 – 0.4347345 × (min(SBP, 110) – 110) /20 + 0.3362658 × (max(SBP, 110) – 130) /20 + 0.7692857 × (if diabetes) + 0.4386871 × (if current smoker) + 0.5378979 × (min(eGFR, 60) – 60) / -15 + 0.0164827 × (max(eGFR, 60) – 90) / -15 + 0.288879 × (if using anti-hypertensive medication) – 0.1337349 × (if using statin) – 0.0475924 × (if using anti-hypertensive medication) × (max(SBP, 110) – 130) /20 + 0.150273 × (if using statin) × ((TC – HDL) × 0.02586 – 3.5) – 0.0517874 × (age – 55) /10 × ((TC – HDL) × 0.02586 – 3.5) + 0.0191169 × (age – 55) /10 × (HDL × 0.02586 – 1.3) /0.3 – 0.1049477 × (age – 55) /10 × (max(SBP, 110) – 130) /20 – 0.2251948 × (age – 55) /10 × (if diabetes) – 0.0895067 × (age – 55) /10 × (if current smoker) – 0.1543702 × (age – 55) /10 × (min(eGFR, 60) – 60) / -15  **Risk** = exp(log-Odds) / (1 + exp(log-Odds)) |


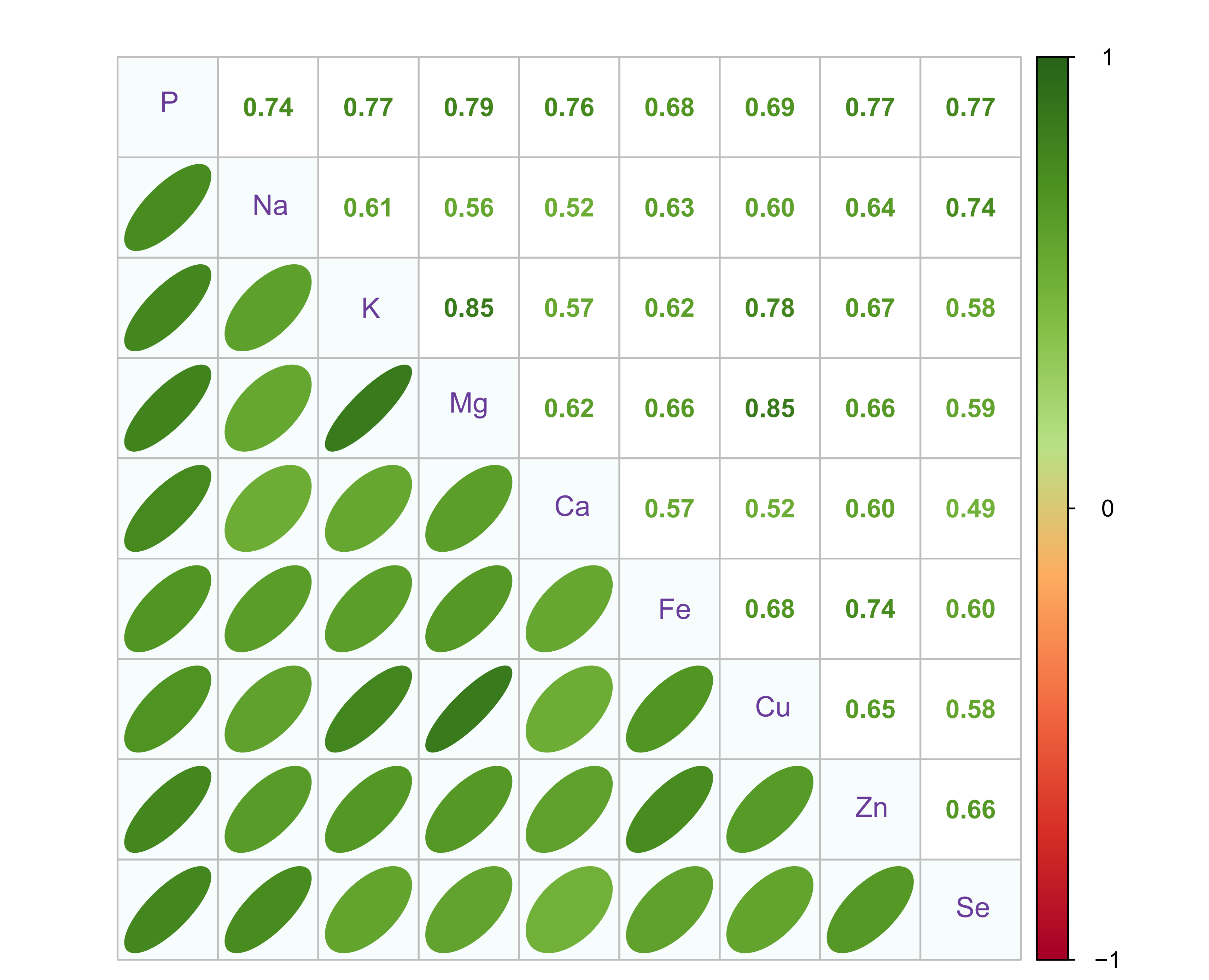


Supplement Figure 1. Correlation heatmap of 9 metal intakes.


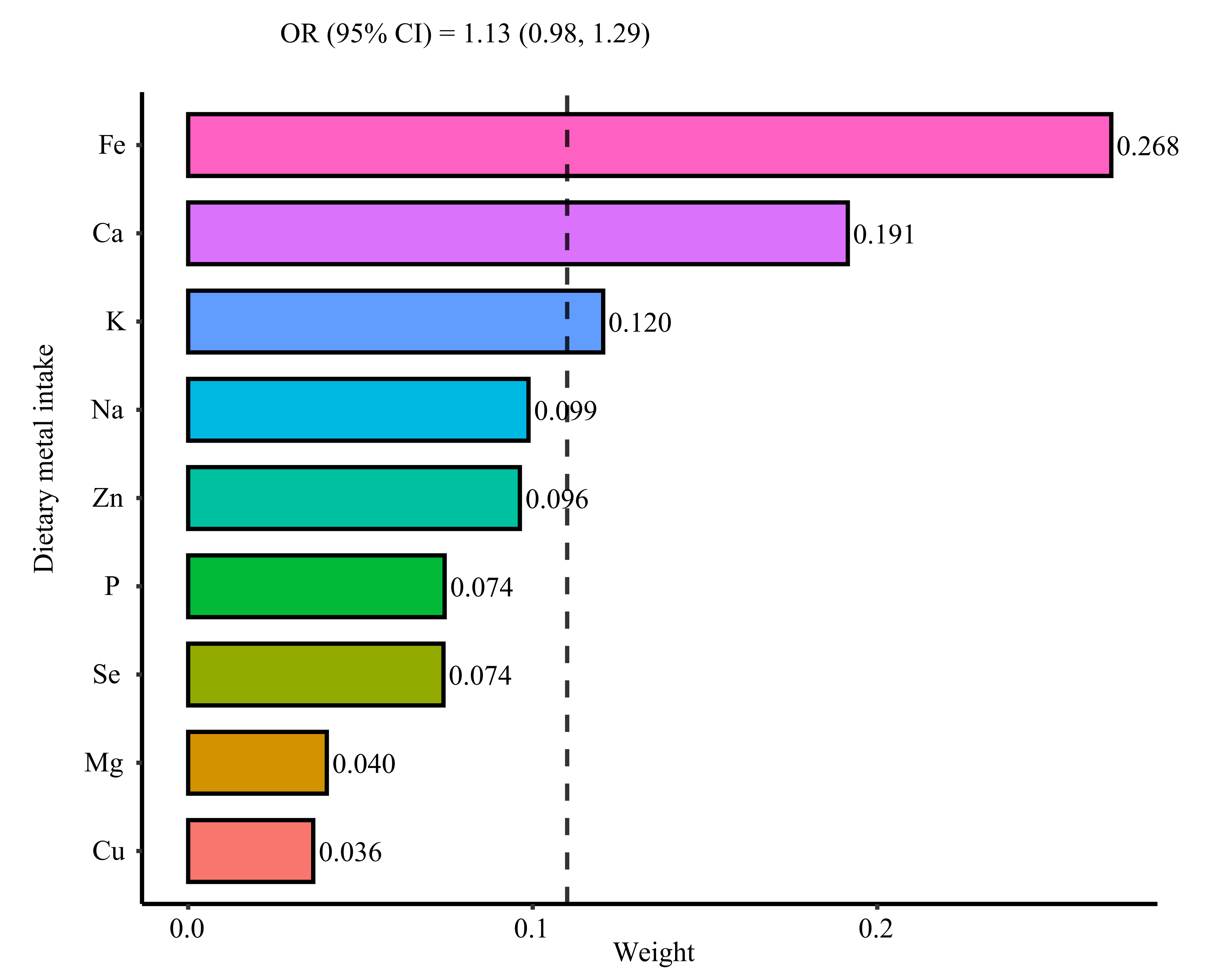


Supplement Figure 2. WQS regression model when hypothesizing a positive relationship between metal intake mixture and advanced CKM stages.
